# Predicting hepatic failure with a new diagnostic technique by preoperative liver scintigraphy and computed tomography: a pilot study in 123 patients undergoing liver resection

**DOI:** 10.1186/s13037-017-0143-z

**Published:** 2017-12-18

**Authors:** Naokazu Chiba, Motohide Shimazu, Kiminori Takano, Go Oshima, Koichi Tomita, Toru Sano, Masaaki Okihara, Yosuke Ozawa, Kosuke Hikita, Takahiro Gunji, Yuta Abe, Kiyoshi Koizumi, Shigeyuki Kawachi

**Affiliations:** 1 Department of Digestive and Transplantation Surgery, Tokyo Medical University Hachioji Medical Center, Tokyo, Japan; 2 Department of Surgery, Tama Kyuryo Hospital, Tokyo, Japan; 3 Department of Surgery, Keio University school of Medicine, Tokyo, Japan; 4 Department of Radiology, Tokyo Medical University Hachioji Medical Center, Tokyo, Japan; 5 1163 Tatemachi, Hachioji, Tokyo 193-0998 Japan

**Keywords:** Remnant liver LU15, Hepatic failure, ^99m^Tc-labelled galactosyl human serum albumin liver scintigraphy

## Abstract

**Background:**

A novel index, total liver LU15, has been identified as a surrogate marker for liver function. We evaluated the ability of preoperative remnant liver LU15 values to predict postoperative hepatic failure.

**Methods:**

Preoperative risk factors for postoperative hepatic failure and remnant liver LU15 were evaluated in 123 patients undergoing liver resection for several diseases from September 1st, 2007 to December 1st, 2016. We calculated the remnant liver LU15 value from the total liver LU15 value and the functional remnant liver ratio. Risk factors for postoperative hepatic failure was determined by univariate and multivariate analysis.

**Results:**

Hepatic failure grade B/C developed postoperatively in six patients of seven patients within Makuuchi criteria / without criteria for remnant liver LU15. Operative time (*p* = 0.0242) and criteria for remnant liver LU15 (*p* = 0.0001) were prognostic factors for hepatic failure according to the univariate analysis. And criteria for remnant liver LU15 (*p* = 0.0009) was only prognostic factor by multivariate analysis.

**Conclusion:**

Based on the findings form this pilot study, it appears that patients with a remnant liver LU15 value of 13 or less may have a high risk of postoperative hepatic failure.

## Background

Before a hepatectomy, it is necessary to evaluate liver function and estimate the function of the future liver remnant. Assessment of hepatic functional reserve is one of the most important issues in hepatic resection [1–4]. This is especially true for patients with both hepatocellular carcinoma (HCC) and liver cirrhosis, or both hilar cholangiocarcinoma and jaundice, that often need an extended hepatectomy. To avoid a resection that would likely lead to postoperative hepatic failure, various methods have been developed for the preoperative assessment of liver function. However, some of these are complex and require calculations involving multiple formulas. To estimate the hepatic functional reserve, ^99m^Tc-labelled diethylene triamine pentaacetate–galactosyl-human serum albumin (^99m^Tc-GSA), a radiopharmaceutical that binds specifically to the hepatic asialoglycoprotein receptor (ASGP-R) has been developed and used clinically to estimate hepatic function [5, 6]. Because ASGP-R is a natural superficial antigen of viable hepatocytes, the uptake of ^99m^Tc-GSA is independent of biochemical processes and allows direct estimation of the functioning hepatocyte mass [7]. In addition, the distribution of ^99m^Tc-GSA in the liver is not dependent on liver blood flow [8]. Koizumi et al. reported that several parameters for ^99m^Tc-GSA liver scintigraphy were estimated and that LU3 and GSAR15 were interesting and promising parameters for assessing liver function [9].

When a hepatic resection involves more than two sections, evaluation of future functional reserve by using 99mTc-GSA and computed tomography (CT) combined fusion images is currently supposed to be more accurate than a combined estimation using an indocyanine green (ICG) retention test and CT [10]. In this study, remnant liver LU15, which was considered the remnant hepatic functional reserve, was estimated using ^99m^Tc-GSA single-photon emission computed tomograpic (SPECT) scintigraphy and CT fusion images. In addition, the functional reserve of the future liver remnant was also estimated before surgery by using fusion images obtained from contrast-enhanced CT and ^99m^Tc-GSA SPECT, and was used to predict liver failure after a hepatic resection. A retrospective study was performed to determine whether the remnant liver LU15 value could enable a final decision regarding a hepatectomy in order to avoid postoperative hepatic failure.

## Methods

### Patients

From September 1st, 2007 to December 1st, 2016 after a ^99m^Tc-labelled galactosyl-human serum albumin liver scintigrapy, a liver resection without biliary tract reconstruction was performed in 123 patients in our department, including 72 patients with HCC, 12 with cholangiocarcinomas, and 35 with metastatic cancers (Table 1). A preoperative portal vein embolization was performed in 21 patients, because their future remnant liver volume was expected to be too small. Liver pathology was classified according to the International Association for the Study of the Liver [11], and the surgical resections were performed according to the liver anatomy of Couinaud [12].

**Table 1:** Patients characteristics

	No. of patients (*n* = 123)
Gender ratio M:F	M:F = 87 : 36
Median age (range)	70 (23–85)
Disease	
HCC	72
CCC	12
Metastatic	35
Others	4
Child-Pugh grade A:B	116:7
Portal vein occlusion (+)	21
Operation procedure	
Partial resection	15
Segmentectomy	18
Mono-sectionectomy	31
Hemihepatectomy	53
Tri-sectionectomy	6
ICG_15_R	12.1 (0.3–30.7)
Makuuchi criteria	
Within	96
Without	27
Parameter from liver scintigraphy	
LHL15	0.918 (0.703–0.977)
HH15	0.623 (0.419–0.906)
LU15	26.7 (10.3–43.5)
remnant liver LU15	17.0 (3.7–43.2)
Criteria for remnant liver LU15	
Within	110
Without	13
Postoperative hepatic failure	
grade B	5
grade C	2

*HCC* Hepatocellular carcinoma, *CCC* Cholangiocellular carcinoma

Preoperatively, each patient was evaluated using ^99m^Tc-GSA scintigraphy, conventional liver biochemistry tests, Child-Pugh grading, and a 15-min retention rate of indocyanine green (ICGR15) test, and the liver volume was measured from CT scans.

### Calculation of remnant liver LU15

**Fig. 1:**
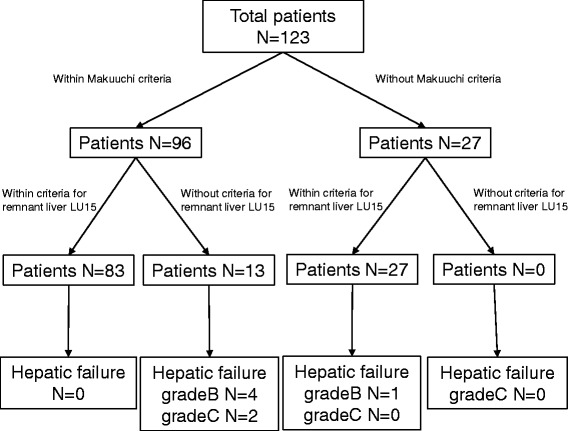
Distribution of patients according to Makuuchi criteria and remnant liver LU15 criteria. All 123 patients were distributed into 96 patients within Makuuchi crietaria and 27 patients without Makuutchi criteria. And 13 patients of 96 patients developed without remnant liver LU15 criteria and all 27 patients without Makuuchi criteria were developed within remnant liver LU15 criteria

All the patients received 3 mg of ^99m^Tc-GSA (185 MBq; Nihon Medi-Physics, Nishinomiya, Japan) as a bolus injection into the antecubital vein. Total liver LU15 was calculated from the cumulative liver uptake of the tracer 15 to 16 min after an injection of a radiotracer. The hepatic SPECT images were acquired after the dynamic study. Data analyses were performed by creating a ROI of the liver and heart and then by drawing their time-activity curves. Each set of projection data was obtained in a 128 X 128 matrix, and 72 projections were acquired. Total LU15 represents the percentage of the integral cumulative count in the liver for 1-min period from 15 to 16 min after tracer injection to total injected dose. Remnant liver LU15 was calculated as an index or residual liver function by applying the following equation; Remnant liver LU15 = Total LU15 x residual count ratio. In the recent cases, with the use of the SPECT images by scintigraphy and a three-dimensional (3D) image of the liver constructed by Synapse Vinscent (FujiFilm, Japan), the regional LU15 of the predicted remnant liver (remnant liver LU15) was calculated according to the operative procedures (Fig. 1). Remnant liver LU15 values of 13.0 and more were considered to represent a remnant liver with good liver function, according to Koizumi et al. [9, 13].

### Postoperative hepatic failure

Postoperative hepatic failure was defined according to the International Study Group of Liver Surgery (ISGLS) classification [14]. The group also differentiated the severity of postoperative liver failure into three different grades from A to C, depending on the level of treatment needed. In this study, grade B and C was defined postoperative hepatic failure. Grade B suggests the degree of liver failure needed clinical management but not invasive therapy; grade C is acute postoperative liver failure requiring invasive treatment [14].

### Operative procedure

The operative procedures included the following; partial resection, segmentectomy, monosectionectomy (eg, right anterior sectionectomy, bisegmentectomy), hemihepatectomy (eg, right or left hepatectomy) and trisectionectomy (eg, right or left trisectionectomy), according to the Brisbane Nomenclature from the International Hepato-Pancreato-Biliary Association [15].

### Statistical analysis

The Mann–Whitney U test was used to examine differences in the laboratory test results. The Fisher’s exact test or the χ^2^ test was used to compare categorical data between the groups. Correlations between the remnant liver LU15 criteria and the other laboratory test results were determined using the Pearson’s correlation coefficient. Odds ratios were used to estimate the relative risk of postoperative hepatic failure. Logistic regression was used for a univariate analysis, while a multiple logistic regression analysis was used for multivariate analysis. For a multivariate analysis, variables with significant (*p* < 0.05) by the univariate analysis were evaluated. *P* values of less than 0.05 were considered significant.

## Results

The mean age of the patients was 70 years, with a range of 23 to 85 years. There were 87 (71%) male and 36 (29%) female patients, yielding a male:female ratio of 2:1; the patients’ Child-Pugh grades included A (94%) and B (6%). The surgical procedures consisted of a partial resections (15), segmentectomy (18), mono-sectionectomy (31), hemihepatectomy (53), and tri-sectionectomy (6) (Table 1). In 21 patients with portal vein occlusion, remnant liver volume increased from 490 g to 607 g.

Ninety six patients within Makuuchi criteria and 27 patients developed without Makuuchi criteria (Fig. 1). 13 patients of all 96 patients within Makuuchi criteria developed without criteria for remnant liver LU15. And 27 patients without Makuuchi criteria were within criteria for remnant liver LU15. Total seven patients developed potoperative hepatic failure over grade B. Table 2 shows the details of seven patients developed postoperative hepatic failure. Two patients developed hepatic failure grade C were performed sgmentectomy, S7/8 segmentactomy and S8 segmentectomy. All two patients died of a hepatic failure while still in the hospital.

**Table 2:** Patients with postoperative hepatic failure grade B/C

Age	Gender	Disease	Child-Pugh	PTPE	ICG_15_R	LU15	Remnant liver LU15	Operative procedure	Postoperative complication
68	F	CCC	A	(-)	9.5	16.7	11.5	Left Hemi.	HF grade B
73	F	HCC	A	(-)	12.3	15.2	12.6	Left Hemi.	HF grade B
68	M	HCC	A	(-)	17.8	14.1	12.1	Left Hemi.	HF grade B
71	M	HCC	A	(-)	15.0	12.6	10.7	S8 Seg.	HF grade B
72	M	HCC	A	(-)	7.2	26.3	12.8	S7/8 Seg.	HF grade C
85	M	HCC	A	(-)	28.5	16.1	12.3	S8 Seg.	HF grade C
73	M	HCC	A	(+)	11.0	31.1	18.8	Right Tri.	HF grade B

*CCC* Cholangio carcinoma, *HCC* Hepatocellular carcinoma, *Hemi.* Hemihepatectomy, *Seg.* Segmentectomy, *Tri.* Trisectionectomy, *HF* Hepatic failure

Table 3 shows the hazard ratios (HR) for the candidate risk factors associated with postoperative hepatic failure as calculated by the univariate and multivariate analysis. Although serum albumin concentration, ICGR15, aspartate aminotransferase (AST), alanine aminotransferase (ALT), and the other factors including the ICGR15 criteria by Makuuchi, et al. were not risk factors, operative time and the criteria for remnant liver LU15 were risk factors for postoperative hepatic failure by univariate analysis. And the criteria for remnant liver LU15 was an only risk factor for postoperative hepatic failure by multivariate analysis.

**Table 3:** Prognostic factor for postoperative hepatic failure

Univariate analysis	Odds ratio	95% CI	*p*-value
Age	1.175	0.950–1.351	0.1239
Sex			
Male	1		
Female	1.024	0.189–5.538	0.9779
Child-Pugh grade			
A	1		
B	2.944	0.304–6.540	0.3514
ICG R15	1.001	0.996–1.007	0.5973
Total billirubin	0.004	0.001–7.003	0.1451
Albumin	0.214	0.019–3.656	0.2870
AST	0.983	0.921–1.049	0.6065
Prothoronbin test	0.946	0.847–1.057	0.3270
Intraoperative blood loss	1.000	0.998–1.001	0.6229
Operation time	1.137	1.017–1.271	0.0242
LHL 15	0.041	0.007–15.070	0.3927
HH 15	0.074	0.009–72.115	0.6485
LU 15	0.965	0.884–1.054	0.9650
Remnant liver LU 15	0.915	0.789–1.062	0.2425
Makuuchi criteria			
Within	1		
Without	1.400	0.257–7.640	0.6975
Criteria for remnant liver LU15			
> 13.0	1		
< 13.0	81.750	8.741–764.566	0.0001
Multivariate analysis			
Operation time	1.073	0.938–1.226	0.3041
Criteria for remnant liver LU15	67.724	6.840–670.561	0.0009

## Discussion

In this study, we attempted to develop a preoperative risk marker for predicting postoperative hepatic failure in patients with any diseases that were candidates for a hepatic resection using 99mTc-GSA scintigraphy. After a bolus intravenousinjection of a radiotracer, sequential anterior abdominal 128 X 128 matrix size images, including the liver and heart, were acquired every 20 s for 20 min. The parameters; HH15, LHL15 and LU15 were calculated from the time-activity curves. Two parameters, HH15 and LHL15, should be used together as complementary indications of liver function, because these parameters seem to reach a plateau value in cases of severe liver dysfunction in HH15 and in cases of liver function improvement in LHL15. In this study, HH15 and LHL15 had week correlation with ICGR15 (data not shown), and the criteria for LHL15 and HH15 were not risk factors for postoperative hepatic failure in univariate analysis. Therefore, HH15 and LHL15 might not be suitable surrogate marker for postoperative hepatic failure. Because LU15 has the advantages of a wider range and a higher value level, as well as better correlation with that of other parameters except ICGR15, LU15 is expected to be the best parameter for regional liver function and seem to be the good surrogate marker for postoperative hepatic failure.

Measurement of the remnant liver volume using planar images obtained by ^99m^Tc-GSA scintigraphy is not accurate, but by creating a cut line in each section of the transaxial or frontal SPECT image, precise measurement of the remnant liver hepatic binding concentration is possible [16]. The amount of remnant liver LU15 could be calculated more correctly before surgery by using fusion images of ^99m^Tc-GSA SPECT and contrast-enhanced CT scans with the Vinsent software. By using these fusion images, any resection area (partial resection, sub-segmentectomy, and any sectionectomy) could be drawn manually on SPECT scans on the basis of the anatomy observed on CT, permitting calculation of the volume of the future liver remnant. Moreover, remnant liver LU15 could be evaluated in cases with obstructive jaundice (for example, hilar cholangiocarcinoma) or a post portal vein embolization, because the hepatic asialoglycoprotein receptor is not affected by jaundice or a portal vein embolization. Thus, radioactivity within the volume of the future liver remnant could be estimated and the remnant liver LU15 values could be calculated.

Many authors have reported methods for assessing liver volume and function using a standard CT, ^99m^Tc-GSA SPECT, or both [17, 18]. In HCC with liver cirrhosis, chronic hepatitis or fibrosis, hepatic dysfunction in the hepatic segment or lobe containing HCC was greater than that of the segments or lobe without HCC because the liver parenchyma around the tumor was damaged by mechanical compression, possibly the result of tumor compression of the vessels and bile ducts. If hepatic CT and SPECT images obtained using ^99m^Tc-GSA were compared, the defect seen on the SPECT images was larger than that of the tumor seen on the CT image. On the other hand, for cases of hilar cholangiocarcinoma with high serum bilirubin levels, we clarified the usefulness of ^99m^Tc-GSA volumetry because evaluation of the ICG test was unreliable, as bilirubin competes with hepatic ICG excretion. Further, a diseased liver without drainage of the obstructive bile duct showed poor functioning. Application of volumetry by ^99m^Tc-GSA scintigraphy might address the limitations of the ICG test, as this test can evaluate separated liver functions in any situation or background liver function [19]. In this study, remnant liver volume estimated by CT volumetry was greater than that of ^99m^Tc-GSA scintigraphy in cases of hilar cholangiocarcinoma (data not shown).

Other studies have evaluated a variety of quantitative liver function tests to predict the risk of postoperative death and complications. Conventional biochemical liver tests, such as serum bilirubin, prothrombin time, and Child-Pugh classification, have only limited value with respect to estimating the hepatocellular reserve. Yamanaka et al. reported that an ICG test, in combination with a radiologic estimation of the liver volume, was of value for predicting liver failure after a hepatectomy [20]. Kinetic analysis of hepatic ICG uptake has been a useful method to evaluate hepatic function, and has been reported to be a good preoperative predictor of death and complications in patients undergoing a liver resection for HCC [21]. The occasional discrepancy between the ICG clearance values and histologic liver findings are thought to depend mainly on the hepatic blood supply and intra/extrahepatic shunt. First, ICG retention is expected to increase in patients with jaundice, because ICG is excreted into the biliary system. Second, a rare constitutional dye excretory disorder has been reported. Moreover, as a test agent, it is well-known that ICG has some adverse reactions such as shock, nausea, rashes and itching. The ability to make a final decision about the extent of liver resection with an underlying liver disease requires new methods to estimate the functional hepatic reserve in the predicted remnant liver, rather than the entire liver.

## Conclusions

Preoperative remnant liver LU15 values independently predicted hepatic failure following a liver resection for any disease, including HCC with liver cirrhosis and hilar cholangiocarcinma with obstructive jaundice. It appears that patients with a remnant liver LU15 value of 13 or less may have a high risk of postoperative hepatic failure.
